# *In silico* transcriptional analysis of mRNA and miRNA reveals unique biosignatures that characterizes different types of diabetes

**DOI:** 10.1371/journal.pone.0239061

**Published:** 2020-09-21

**Authors:** Juan M. Cubillos-Angulo, Caian L. Vinhaes, Eduardo R. Fukutani, Victor V. S. Albuquerque, Artur T. L. Queiroz, Bruno B. Andrade, Kiyoshi F. Fukutani

**Affiliations:** 1 Instituto Gonçalo Moniz, Fundação Oswaldo Cruz, Salvador, Brazil; 2 Faculdade de Medicina, Universidade Federal da Bahia, Salvador, Brazil; 3 Curso de Medicina, Faculdade de Tecnologia e Ciências (FTC), Salvador, Brazil; 4 Multinational Organization Network Sponsoring Translational and Epidemiological Research (MONSTER) Initiative, Salvador, Brazil; 5 Escola Bahiana de Medicina e Saúde Pública (EBMSP), Salvador, Brazil; 6 Universidade Salvador (UNIFACS), Laureate Universities, Salvador, Brazil; Novo Nordisk Foundation Center for Protein Research, University of Copenhagen, DENMARK

## Abstract

Diabetes (DM) has a significant impact on public health. We performed an *in silico* study of paired datasets of messenger RNA (mRNA) micro-RNA (miRNA) transcripts to delineate potential biosignatures that could distinguish prediabetes (pre-DM), type-1DM (T1DM) and type-2DM (T2DM). Two publicly available datasets containing expression values of mRNA and miRNA obtained from individuals diagnosed with pre-DM, T1DM or T2DM, and normoglycemic controls (NC), were analyzed using systems biology approaches to define combined signatures to distinguish different clinical groups. The mRNA profile of both pre-DM and T2DM was hallmarked by several differentially expressed genes (DEGs) compared to NC. Nevertheless, T1DM was characterized by an overall low number of DEGs. The miRNA signature profiles were composed of a substantially lower number of differentially expressed targets. Gene enrichment analysis revealed several inflammatory pathways in T2DM and fewer in pre-DM, but with shared findings such as Tuberculosis. The integration of mRNA and miRNA datasets improved the identification and discriminated the group composed by pre-DM and T2DM patients from that constituted by normoglycemic and T1DM individuals. The integrated transcriptomic analysis of mRNA and miRNA expression revealed a unique biosignature able to characterize different types of DM.

## Introduction

Diabetes mellitus (DM) is a group of chronic metabolic disorders characterized by the elevation of blood glucose levels (hyperglycemia) due to defects in insulin secretion and/or activity [1]. The most recent report from the American Diabetes Association (ADA) indicated that in 2017, approximately 425 million adults were diagnosed with DM and estimated that by 2045, another 629 million people will be afflicted by these metabolic disorders [2]. DM can be broadly classified into two categories: type-1 (T1DM) and type-2 diabetes (T2DM). The former, also known as “insulin-dependent DM”, corresponds to 5–10% of DM cases, and is characterized by insufficient or suboptimal production of insulin as a result of cellular mediated-destruction of the pancreatic beta cells [3]. On the other hand, T2DM, also known as “non-insulin-dependent DM”, accounts for 90–95% of all DM cases. The wide spectrum of symptoms observed in T2DM patients is associated with either insulin resistance or partial insulin deficiency which stems from a variety of causes, including obesity, aging, and sedentarism [4]. Of note, pre-diabetes (pre-DM) is considered a transitional condition to DM in which an individual exhibits elevated blood glucose levels without meeting additional criteria for diagnosis of DM [5]. Furthermore, shreds of evidence gathered in the last years suggest an association between pre-DM and a higher risk for the development of cardiovascular diseases (CVDs) [6]. Despite the current advance surrounding the understanding of differences and similarities in the pathogenesis of distinct types of DM, the underlying molecular mechanisms remain only partially understood.

The study of the role of the key determinants of the DM pathogenesis is important to understand DM as a chronic inflammatory disease and to guide development of better therapeutic approaches [7]. A large number of inflammatory markers have been suggested as pathogenic mediators for DM, including C-reactive protein, interleukin (IL)-1, IL-6, and tumor necrosis factor (TNF)-α [8]. Indeed, the inflammatory biomarkers, many of which are secreted by adipocytes [9]^9^, correlate with prevalent and incident DM as well as its major complications [8]. Of note, several novel biomarkers, biological pathways, and cellular processes have also been associated with DM progression, which includes transcriptional markers and micro-RNA (miRNAs) [10]. miRNAs are a class of small non-coding RNAs, containing 17–25 nucleotides, that are central regulators of gene expression and important players in the development of different forms of DM [11]. Furthermore, miRNAs have been described to regulate pancreatic β-cell development and function and also to modulate the expression of several key genes in tissues that respond to insulin [12]. A large body of evidence indicates that changes in miRNAs levels are associated with a higher risk of developing DM-related outcomes such as renal complications, cardiovascular disease and visual impairment [13].

The investigation of miRNAs and other transcripts as biomarkers of DM pathogenesis has potential to provide a powerful tool for the attainment of a better understanding of such disease. Therefore, we performed an *in silico* study of publicly available datasets of paired mRNA and miRNA transcript expression with the hypothesis that there are combined transcriptomic signatures capable of characterizing distinct biological processes underlying the different types of DM.

## Materials and methods

### Ethics statement

There were no patients directly involved in the research. The present study used publicly available gene expression data from previously published studies to perform a meta-transcriptome analysis. All information given to the research team was de-identified. Thus, the study was exempted from revision by the Institutional Review Board of the Instituto Gonçalo Moniz, Fundação Oswaldo Cruz, Salvador, Brazil, and did not require signed consent forms.

### Description of datasets

**Fig 1:**
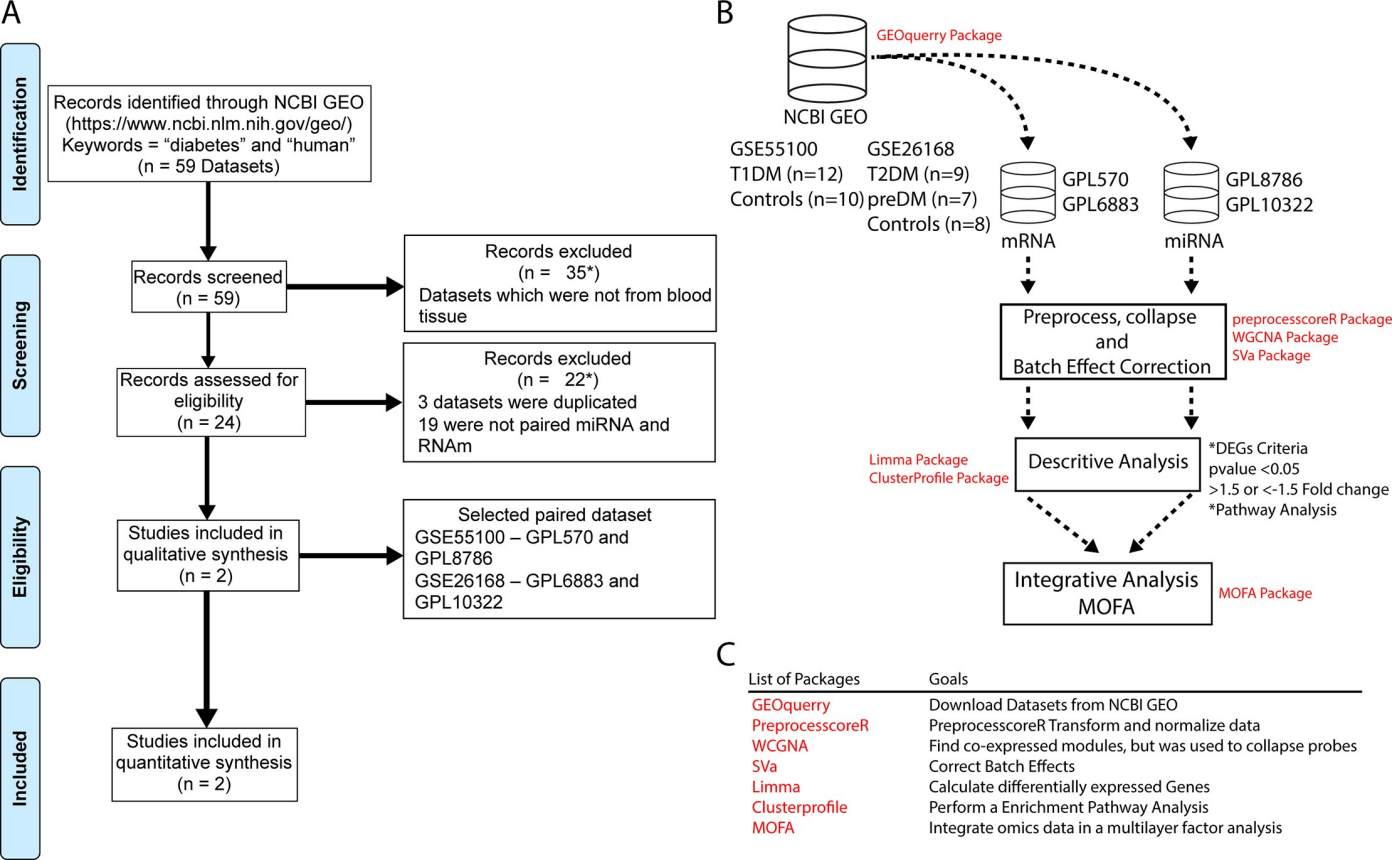
PRISMA flow chart of the transcriptional meta-analysis. (A)Selection of eligible GEO datasets for systems biology analysis according to PRISMA 2019 flow diagram. (B) Flow diagram of bioinformatics analysis. (C) A list of packages and functions.

To select the databases, we accessed the Gene Expression Omnibus (GEO) website in May 28, 2018 [14]. The main objective was to evaluate datasets that contained transcriptional records on expression of both mRNA and miRNA from peripheral blood samples. The following general terms were used: “Diabetes”, and “*Homo sapiens*”. This approach resulted in a total of 59 datasets. We next excluded 35 datasets because those studies used samples from tissues other than blood (Fig 1A). Then, 24 records were examined for eligibility and additional 22 were excluded (3 datasets were duplicated and 19 did not contain paired data on mRNA and miRNA (Fig 1A). Finally, 2 datasets were included from persons with normoglycemia, pre-DM, T1DM or T2DM paired with mRNA and miRNA. The first dataset was GSE55100 which was obtained from experiments using peripheral blood samples from patients with T1DM compared with normoglycemic controls, recruited at the Ruijin Hospital in Shanghai between January 2009 to September 2012 [15]. In this study, a total of 22 samples of 11 males and 11 females individuals were processed and further stratified in10 normoglycemic controls and 12 newly diagnosed T1DM patients. In addition, two microarray platforms were used to obtain the data from the same samples: GPL570 (measured the mRNA) and GPL8786 (measured the miRNA). The second dataset (GSE26168), had data from a total of 60 samples from peripheral blood of male individuals recruited at the Alexandra Hospital, Singapore, between July 2008 to April 2009. The data were generated using two platforms: GPL6883 (to measure mRNA) and GPL10322 (to measure miRNA) [16]. The dataset was composed by 8 normoglycemic healthy controls, 7 individuals with impaired fasting glucose and 9 patients with T2DM. This dataset also contained 10 samples from an animal model (*rat norvegicus*), which were not analyzed in the present study. All the data were downloaded on June 08,2018.

### Data collection and curation of datasets

The datasets from both platforms (mRNA and miRNA) were obtained using the *GEOquerry* package [17] in R 3.6.2 and raw expression data were downloaded. GEOquerry is a package which allows the downloading of all information from the NCBI GEO datasets, including metadata, in R platform. After data acquisition, data were normalized and log2 transformed using the *preprocesscore R* package [18] in R 3.6.2. This package was used to transform and normalize all transcriptomic data. The probes identification in GSE55100 (performed on Affymetrix platform) and GSE26168 (performed on Illumina platform) were corrected by collapse function (*collapserows)* presented in *Weighted correlation network analysis (WGCNA)* package in R 3.6.2. The WGCNA allows the identification of gene coexpression modules, based on the correlation networks. In this analysis, we used the collapsed probes with its annotated gene symbol. The function *collapseRows* was used to summarize the probes into its respective annotation. Thus, the GSE26168 dataset presented 10,899 probes and the GSE5510 dataset 23520. The collapsing process resulted in 8,131 genes and 847 miRNA in the GSE26168 dataset and 3,368 genes and 668 miRNA, respectively in the GSE5510 dataset. We only analyzed the genes and miRNA presented in both datasets, discarding the particular ones, with 7579 genes and 668 miRNA present in both datasets. After collapsing the probes and merging the count tables, the expression data were submitted to a correction procedure of batch effect using an empirical Bayes framework implemented in the *ComBat* function available in *SVApackage [19] in R 3.6.2.* This package is used to correct the batch effects in high throughput data to minimize the experimental variance.

The differentially expressed genes (DEGs) were calculated comparing each clinical group (pre-DM, T1DM and T2DM) with normoglycemic healthy controls (baseline group). Genes were considered differentially expressed when presented log2-fold-change < -1.5 or log2-fold-change > 1.5 and p-value corrected with the Benjamini–Hochberg false discovery rate (FDR) adjustment for multiple testing (FDR = 5%) lower than 0.05, determined with limma package [20]. A volcano plot was used to identify the changes in expression, for this we use -log 10 transformed *p-value* corrected by FDR versus fold-change on the y and x-axes, respectively. Venn diagrams were used to visualize/summarize all possible logical relations of all the DEGs between the clinical groups. Principal component analysis (PCA) was performed in order to estimate the variance of the global gene expression. For this analysis, we applied the function *prcomp* a native package in R 3.6.2. The enrichment pathway analysis was employed to functionally describe the DEGs, using the Kyoto Encyclopedia of Genes and Genomes (KEGG) database v6.2 [21] and *Clusterprofile* packages [22] in R 3.6.2. This approach was employed in order to determine an over-represented gene set in the DEGs also identifying significantly enriched or depleted genes in this set. The *Clusterprofile* algorithm performed an enrichment pathway analysis and information is available in: https://bioconductor.org/packages/release/bioc/html/clusterProfiler.html

### Multi-omics factor analysis

Multi-omics factor analysis (MOFA) enables the analysis of biological multidimensional data, ranging from genome, epigenome, transcriptome, proteome, and metabolome, integrating all these layers across a more comprehensive result in a latent factor [23]. The MOFA model used here integrated data from mRNA and miRNA was defined by several parameters. First, the logical scale and paired samples were selected by the functions #scaleViews (recommended to be “FALSE” for small datasets) and #removeIncomplete (was set as “TRUE” to only use the paired samples in both datasets of mRNA and miRNA). Second, the number of factors was selected by default (value = 0.5), meaning that the model would only remove a factor if it explained exactly zero variance in the data. In summary, this parameter shows all the factors. The data on factors was next processed with the function #likelihood = “gaussian”, which fits the model according with the data. Gaussian for continuous data, Bernoulli for binary data and poisson for count data. Furthermore, the parameter #sparsity was set as “TRUE” to automatically learn the appropriate level of regularization for each factor and improve the interpretation. Third, the tolerance was set as 0.01, recommended to establish a model and restrict the number of factors. After selecting all the parameters and preparing the datasets, we used as an input the corrected bath effect count table of the mRNA and miRNA obtained as the formal analytical merged dataset. All the data were paired by study, individual and platform. In all analyses, ap-value < 0.05 after the 5% FDR adjustment was considered statistically significant. All the default pipelines of the analyses performed here are available in: http://www.bioconductor.org/packages/devel/bioc/html/MOFA.html. The overall analytical study design is illustrated in Fig 1B and a list of packages are provided in Fig 1C.

## Results

### Patients with different types of DM exhibit a distinct profile of mRNA and miRNA expression

Before formally initiating the transcriptome analysis, we checked the batch effects in the preprocessed data. The distribution of the patients in the setting of uncorrected data demonstrated a large batch effect between platforms and experiments (S1A and S2A Figs). After the correction, the variance was reduced in both mRNA (60.7% to 20.9%) and miRNA (85.6% to 9.4%); and the individuals from control groups in both datasets of mRNA and miRNA were colocalizated in the same space of the principal component analysis (PCA) plots. This approach also indicated that biological sex was not a parameter which influenced the distribution of individuals in the PCA plots (S1B and S2B Figs). We then utilized the data after correction of the batch effect in the further steps of the investigation.

**Fig 2:**
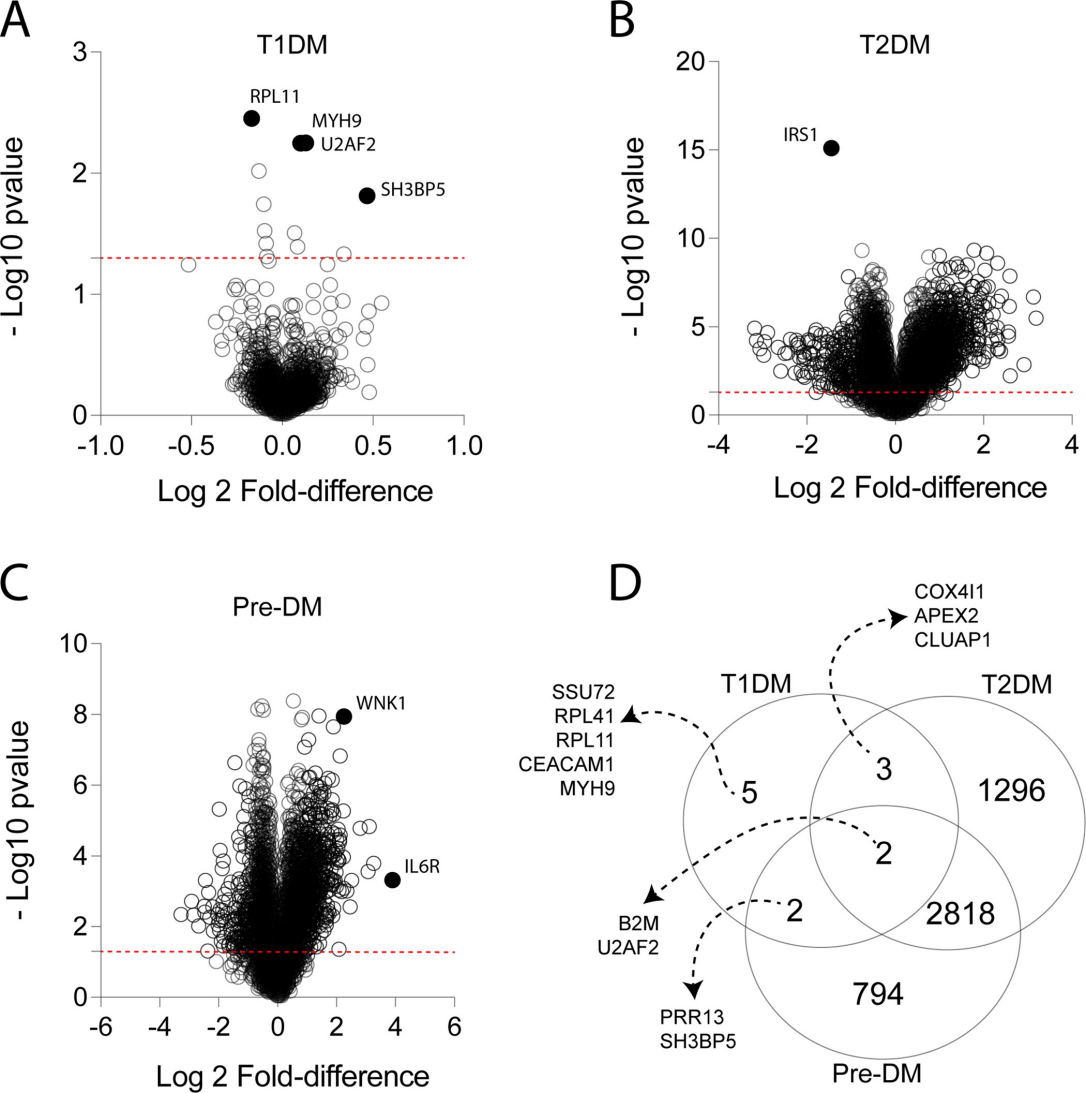
Differentially expressed mRNA transcripts among individuals with different types of dysglycemia. Volcano plots display the differentially expressed genes based on adjusted p-value and fold-difference variation of the gene expression in comparisons between each clinical group and the reference group (normoglycemic healthy controls) as follows: T1DM **(A)**, T2DM **(B)** and pre-DM **(C)**. In **(D)**, Venn diagram shows genes with significant adjusted p-values (<0.05) of each comparison between the indicated groups and the reference group using the Student’s T-test. Details of all the comparisons are available in the S1 File.

In the first comparisons, we investigated the differentially expressed transcripts detected in the mRNA datasets (Fig 2). The criteria for DEGs was established as log2 fold-change >1.5 or <-1.5 and p value <0.05. Patients with T1DM exhibited a total of 12 genes with significant p-values, but all of those displayed low values of fold-change variation compared to normoglycemic healthy controls, failing in the criteria (Fig 2A). On the converse, patients with T2DM had a high number of genes with significant p-values (n = 4119) with 159 genes on the criteria (Fig 2B). Individuals with pre-DM had 3616 significant genes and 143 of those genes met the fold-change criteria (Fig 2C). We next used a Venn diagram of all those genes identified within the criteria between all clinical groups (pre-DM, T1DM and T2DM) to summarize the findings. The Venn diagram indicated that there was a high number of transcripts which were uniquely expressed in the groups of individuals with pre-DM (n = 794) or T2DM (n = 1296), whereas patients with T1DM exhibited only 5 uniquely expressed genes (*SSU72*, *RPL41*, *RPL11*, *CEACAM1* and *MYH9*) (Fig 2D). In addition, while a total of 2818 transcripts with significant p-values was found in exclusively between the pre-DM and T2DM groups, only 2 (*PRR13* and *SH3BP5*) genes were found in common between pre-DM and T1DM and 3 (*COX4I1*, *APEX2* and *CLUAP1*) were detected between T1DM and T2DM (Fig 2D). Of note, only 2 genes with significant p-values (*B2M*, *U2AF2)* were found to be in common among all the three clinical groups (Fig 2D).

**Fig 3:**
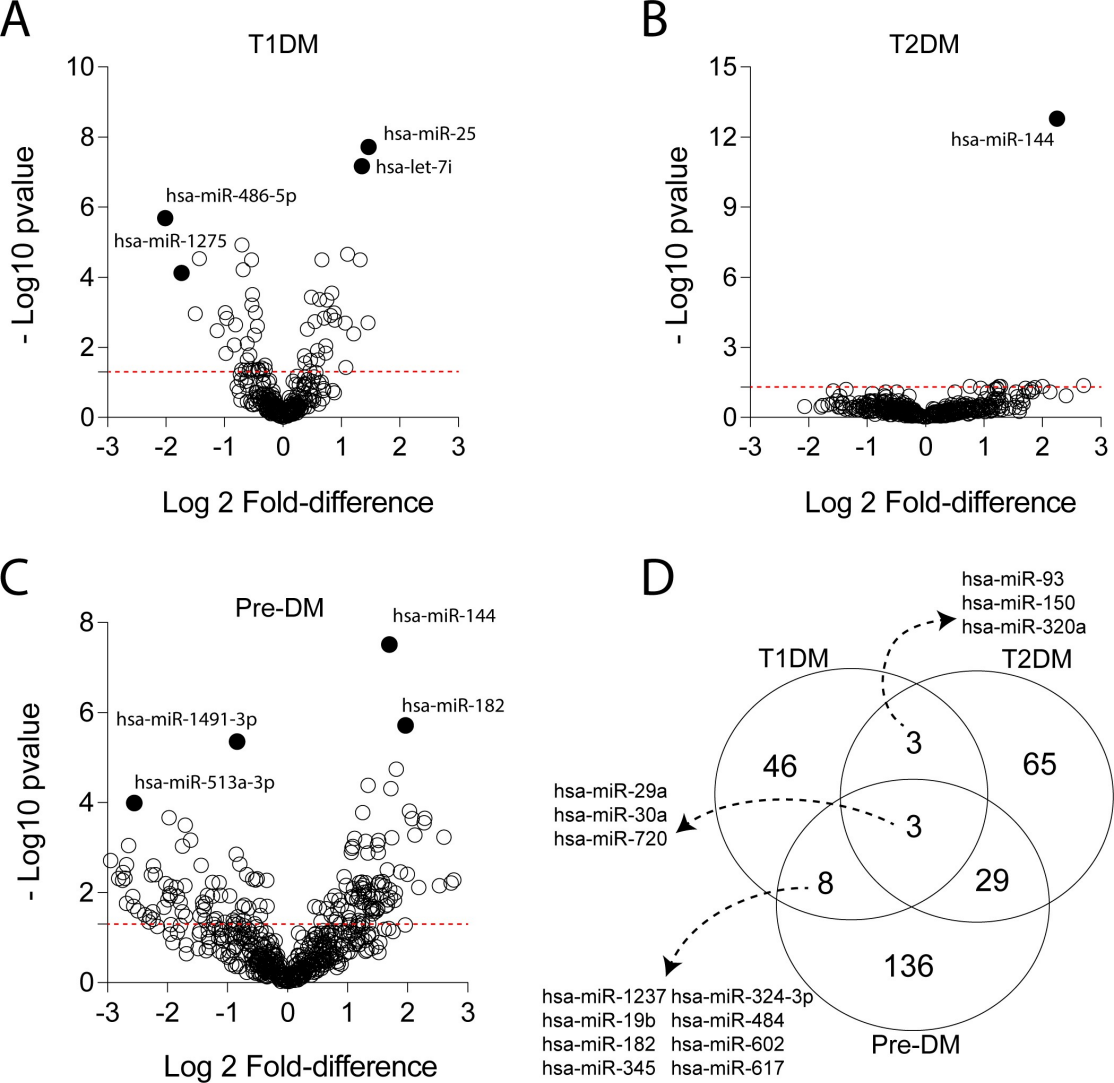
Differentially expressed miRNAs among individuals with different types of dysglycemia. Volcano plots display the differentially expressed miRNA based on adjusted p-value and fold-difference variation of the miRNA expression in comparisons between each clinical group and the reference group (normoglycemic healthy controls) as follows: T1DM **(A)**, T2DM **(B)** and pre-DM **(C)**. In **(D)**, Venn diagram shows the miRNA with significant adjusted p-values (<0.05) of each comparison between the indicated groups and the reference group using the Student’s T-test. Details of all the comparisons are available in the S1 File.

With regard to miRNA expression, T1DM individuals displayed 60 transcripts with significant p-values and only three matching the DEGs fold-change criteria (hsa-miR-486-5p, hsa-miR-1275 and hsa-miR-744) (Fig 3A). The group of patients with T2DM displayed 100 different miRNAs which were statistically significant but just one with fold-difference value over the control group (hsa-miR-144) (Fig 3B). Moreover, pre-DM individuals presented 176 miRNAs with significant p-values and 82 targets matched the DEGs criteria (Fig 3C). A Venn diagram was once again used to depict the relations in transcript expression between the groups. Similarly to what we found in the analysis of the mRNA expression, the diagram with the miRNA data revealed a relatively high number of statistically significant transcripts exclusive to pre-DM (n = 136) and to T2DM (n = 65) whereas the T1DM group exhibited the lowest number of uniquely expressed miRNA (n = 46) (Fig 3D). Furthermore, while 29 miRNAs were found in common between pre-DM and T2DM, only 8 were detected among pre-DM and T1DM and 3 were observed exclusively between T1DM and T2DM (hsa-miR-93, hsa-miR-150 and hsa-miR-320a) (Fig 3D). Three miRNAs with significant p-values were shared by the three clinical groups (hsa-miR-29a, hsa-miR-30a and hsa-miR-720).

### Pathway enrichment analysis of mRNA transcripts revealed unique profiles in pre-DM and T2DM, but not in T1DM

**Fig 4:**
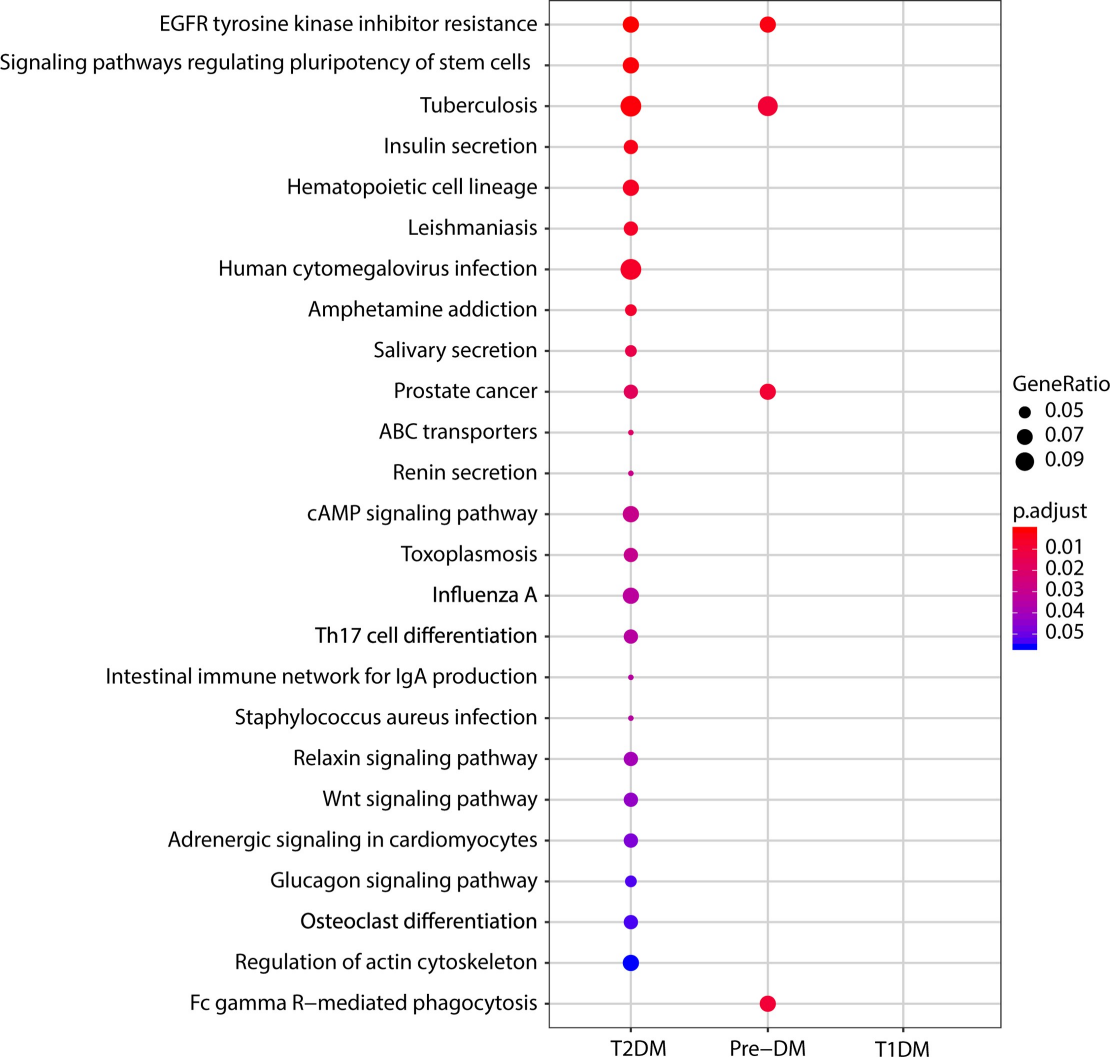
Pathway Enrichment Analysis of the differentially expressed genes per each clinical group. The differentially expressed genes (DEGs) were analyzed using a pathway enrichment compared to the KEGG database as described in the Methods section. Only statistically significant enriched pathways are depicted. No pathway was observed in the T1DM group because there were no DEGs identified.

To understand the pathways associated with different types of DM, we employed a pathway enrichment analysis using the DEGs compared to the KEGG database. Interestingly, there was no enriched pathway detected in the T1DM group because of the complete lack of DEGs in the primary analysis. On the converse, in T2DMwe found several pathways, including “Tuberculosis”, “human cytomegalovirus infection” and “Th17 cell differentiation” (Fig 4). The group of individuals with pre-DM displayed 4 enriched pathways. Of those, the “EGFR tyrosine kinase inhibitor resistance”, “prostate cancer” and, interestingly, “Tuberculosis” were shared in common with T2DM (Fig 4). One pathway (“FC gamma R-mediated phagocytosis”) was exclusively represented in pre-DM.

### The Integration of mRNA and miRNA data identified factors able to distinguish pre-DM and T2DM fromT1DM and normoglycemic healthy controls

**Fig 5:**
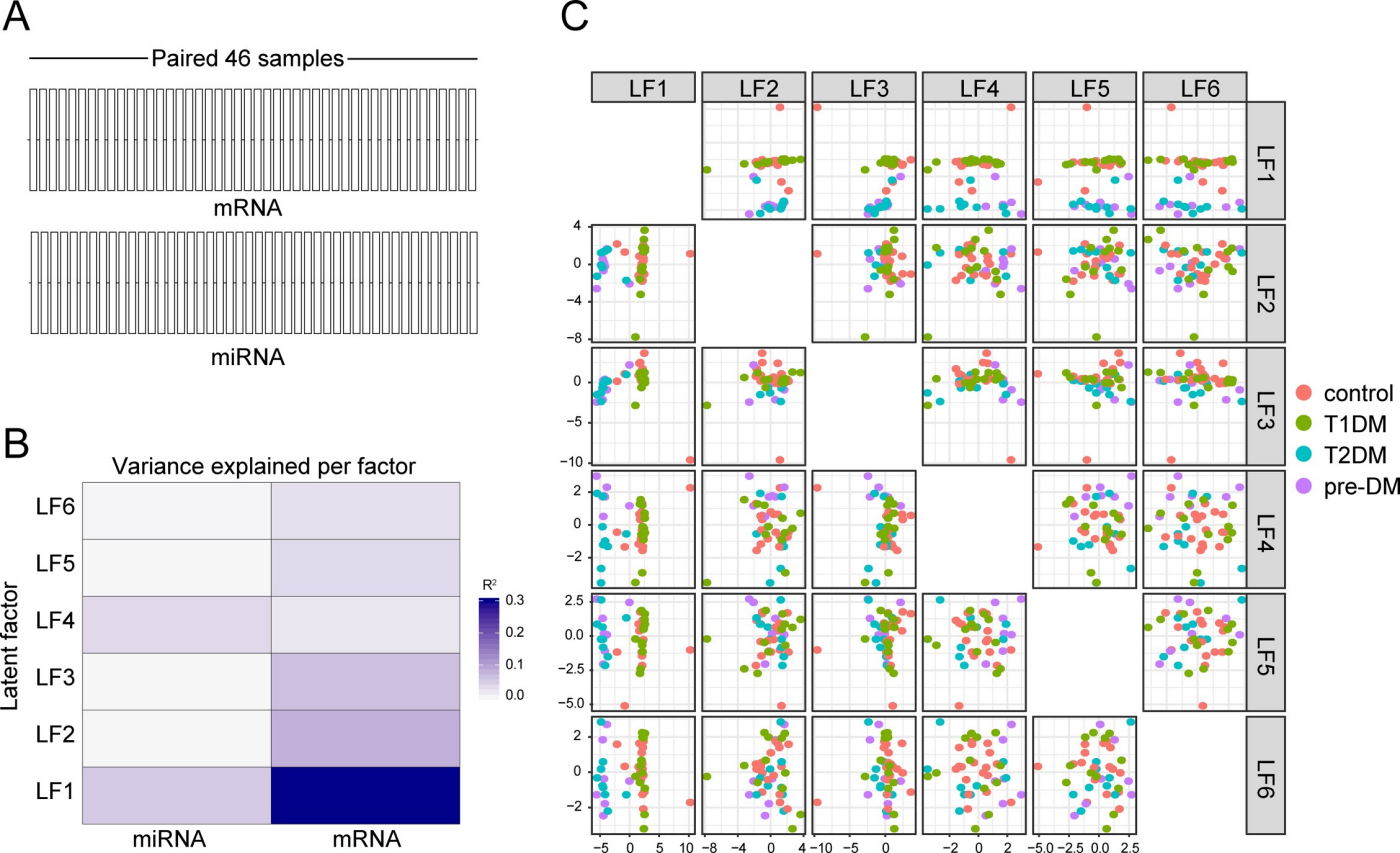
Multi-Omics Factor Analysis identified latent factors able to distinguish pre-DM and T2DM from T1DM and normoglycemic healthy controls. (A) 46 samples with paired data on mRNA and miRNA expression were used using MOFA as described in Methods. (B) MOFA summarized the mRNA and miRNA data in 6 latent factors (LF) with different associations (evaluated using the proportion of total variance explained, R^2^) with the mRNA dataset, the miRNA dataset or both. (C) Each latent factor was inputted as a principal component in a PCA algorithm and a matrix was used to show different combinations of latent factors able to segregate the distinct clinical groups.

After performing analyses using the datasets and describing the lack difference in mRNA expression in patients with T1DM and differences in miRNA expression separately, we performed an integrative multi-omics factor analysis to investigate two transcriptomics layers of the same patients using MOFA pipeline [23]. To do that, a total of 46 samples, which had full pairing between mRNA and miRNA available data were used (Fig 5A). The MOFA integrated the omics in 6 latent factors. Among those, the latent factor 1 exhibited the highest influence in the discriminantion between the clinical groups and it was strongly correlated with the transcriptomics of mRNA and miRNA, whereas the latent factor 4 was more associated with the miRNA data (Fig 5B). We next used a PCA model in which each latent factor was inputted as a principal component, to identify potential combined factors that could cluster the different clinical groups separately. This approach revealed that the latent factor 1 and 4 together resulted in reliable capacity to discriminate the groups, when combined. The factor 1 was able to more consistently separate the groups of pre-DM patients and T2DM from those of T1DM and normoglycemic controls (Fig 5C).

### The latent factor is related with several candidate markers of distinct types of DM and enriched in pathways related to homeostasis

**Fig 6:**
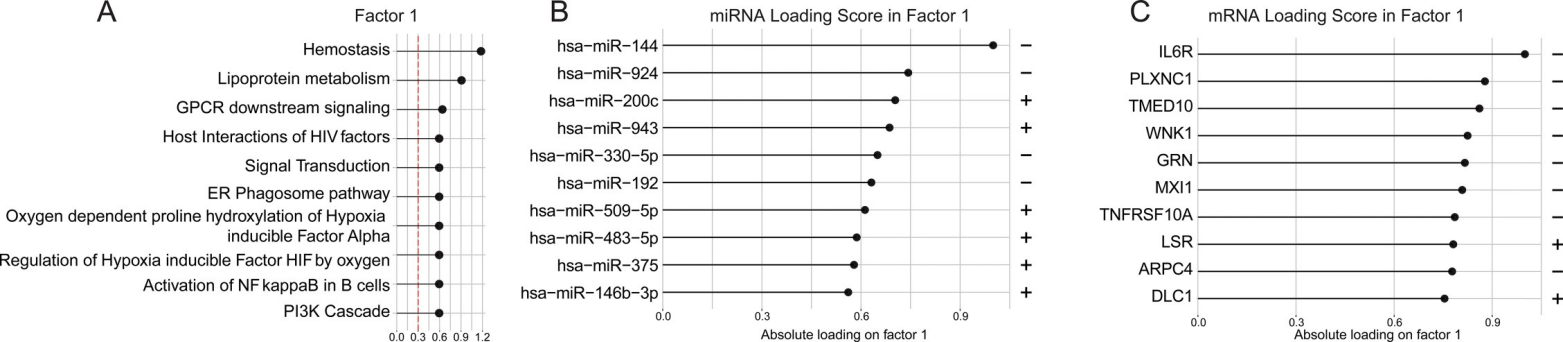
Functional analysis of loading values from latent factor 1. **(A)** Pathway enrichment analysis of the latent factor 1. **(B)** Loading values miRNA composing the latent factor 1. **(C)** Loading values mRNA composing the latent factor 1. In **(B)** and **(C)** the “+” and “-” signs infer directionality of the influence of the distribution of data points shown in Fig 4C.

The results presented above using the MOFA approach identified two latent factors (LF1 and LF4) that when combined could result in better separation of the subgroups of individuals from the different clinical groups. The LF1 was the factor that exhibited the strongest discriminatory potential (Fig 5C). We then used the information on the loading score values of each marker that constituted LF1 to describe the most relevant factors driving the separation of the clinical groups. The MOFA package includes a functional enrichment analysis of pre-defined biological pathways based on the results of the loading scores from the latent factor [23]. Our results demonstrated that the LF1 was most significantly enriched for “hemostasis”, followed by and “GPCR downstream signaling”, while being less significantly associated with “regulation of hypoxia inducible factor HIF by oxygen”, “activation of NF-kappaB in B cells” and “PI3K cascade” (Fig 6A).

The different latent factors are uniquely influenced by the loading scores from the distinct markers (miRNA and mRNA) [23]. To better delineate which markers were influencing more substantially the LF1, we plotted the loading scores from the miRNA and mRNA datasets. In this analysis, increases in the transcript loading values are proportional to increases in association with the latent factor. In addition, the direction of the effect is illustrated by “+” (positive loading values) or “-” (negative loading values). Following this approach, we found that miRNAs that more strongly contributed to the LF1 were miR-144 and miR-924 (Fig 6B). Of note the loading values of these two miRNAs were negative, indicating that they are more associated with T2DM and pre-DM patients because those clinical groups had negative values of the LF1 as shown in Fig 6C. On the converse, miR-200c and miR-943 exhibited positive loading values (Fig 6B), which were associated with normoglycemic healthy controls and individuals with T1DM (Fig 4C). The same rational was employed with mRNAs, where *IL6R*, *PLXNC1*, *TMED10*, *WNK1*, *GRN*, *MXI1*, *TNFRSF10A* all displayed negative values and thus being related toT2DM and pre-DM patients, whereas *LSR* and *DLC1*had positive values (Fig 6C) and were then related to normoglycemic controls and T1DM (Fig 5C).

## Discussion

In the present study, we analyzed publicly available transcriptomic datasets containing information on both mRNA and miRNA expression levels from peripheral blood of individuals with pre-DM, T1DM and T2DM as well as from normoglycemic controls using innovative systems biology tools, such as MOFA. Most of the previous studies in the field evaluated mRNA and miRNA datasets individually in the context of DM [24,25]. Our integrative analysis identified combined molecules which could explain at least in part the dissimilarities between the group of patients with pre-DM or T2DM and the group of T1DM and normoglycemic controls. Such molecules included both specific miRNA and mRNA transcripts which created a latent factor with a discriminant capacity in the MOFA results. These findings are important to help understand the possible molecular differences among T1DM, T2DM and pre-DM.

In the primary analysis, we examined the differentially expressed transcripts detected in the mRNA and miRNA datasets studied individually. Such approach demonstrated that, when compared with normoglycemic controls, patients with TD1M exhibited the lowest number of uniquely expressed transcripts than the patients from the groups of T2DM and pre-DM. Of note, genetic differences between T1DM and T2DM, which may have implications on the transcriptional profiles, have been previously reported [26]. For example, a genome-wide association study revealed that approximately one-third of genetic determinants of diabetes are associated with T2DM, but not with T1DM [27]. In addition, a comparative analysis of mRNA expression in the peripheral blood has identified that the differential gene expression was higher in patients with T2DM compared to T1DM patients, suggesting that T2DM is more frequently associated with activation of pathogenetic pathways [28]. Patients with T1DM are insulin dependent and all of the patients included in the analyses presented here were undergoing insulin treatment for 6 months or more [15]. Insulin therapy has been reported to have a direct impact in the gene expression levels, normalizing the majority of the genes which are dysregulated in response to diabetes, including those involved with inflammatory processes, microvascular integrity, and neuronal function in euglycemic diabetic rats [29]. Finally, in regard to data on miRNA, patients with T1DM have been shown to exhibit decreased expression levels of miR-150, miR-146a and miR-424 compared to T2DM patients [30]. The body of work described above argues that the transcriptional profile of T2DM is more perturbed than that observed in T1DM. Additional studies will be necessary to define the molecular mechanisms underlying this phenomenon.

The pathway enrichment analysis of the differentially expressed genes revealed that “EGFR tyrosine kinase inhibitor resistance” was shared in common between T2DM and pre-DM. Some studies using experimental mouse models reported that epidermal growth factor receptor (EGFR) mRNA expression is down-regulated in pancreatic islet cells and related to onset of diabetes [31]. On the converse, findings from another study examining murine mesangial cells demonstrated a beneficial effect of EGFR inhibition against cell death mediated by EGFR which preceded development of diabetes [32]. Whether the EDFR-related pathway has direct implication in development of diabetes in humans has not been formally demonstrated. The pathway “Tuberculosis” was also shared in common with T2DM and pre-DM. Importantly, strong epidemiological and pathophysiological evidence support the idea that T2DM is a major risk factor for the development of active pulmonary tuberculosis (TB) [33]. It has been recently demonstrated that patients with high levels of glycated hemoglobin (HbA1c) exhibit increased risk of TB in Sudan [34]. Furthermore, a study in India reported that individuals with pre-DM and latent TB infection (LTBI) have a high risk of developing active TB [35]. Finally, DM is associated with altered levels of specific cytokines and chemokines that affect adaptive immunity that are potentially relevant to anti-TB immune responses [33]. Lastly, the pathway “FC gamma R-mediated phagocytosis” was exclusively represented in the group of individuals with pre-DM. To our knowledge, Fc-gamma receptor has not previously been reported to be associated with pre-DM, nevertheless some studies suggest an association between autophagy and pre-DM. Indeed, a study suggested that mice genetically lacking genes that promote autophagy exhibit early onset of altered glucose metabolism related to a profile similar to pre-diabetes [36]. Furthermore, an investigation of 127 non-diabetic individuals found a clinically relevant decrease in the phagocytic index laboratory results which was proportional to increasing blood glucose levels [37]. Although interesting insights on the pathogenesis of pre-DM and T2DM could be developed using the pathway enrichment analysis, no advance could be gained from the analysis of T1DM, because there was no differentially expressed mRNA in our primary analysis, which preclude inferences in pathways involved. Thus, additional studies using a larger number of patients with T1DM are necessary to delineate specific pathways associated with this condition.

In recent years, several studies have explored the use of miRNA expression data in blood as diagnostic and prognostic biomarkers in the context of several diseases [38]. miRNAs are central regulators of gene expression and thought to be important players in the development of pre-diabetic and diabetic conditions [11]. Pancreatic β-cells and the tissues targeted by insulin express a well-defined set of miRNAs [39]. Our study indicates miR-144, miR-924, miR-943 and miR-200c were the miRNAs which most contributed to the latent factor 1, that in turn could separate the groups of T2DM or pre-DM from those of normoglycemic controls or withT1DM. Our results are in agreement with a previous study which found that miR-144 expression is highly up-regulated in T2DM [16]. In this context, increased expression of miR144 is linked to impaired signaling of the insulin cascade probably due to the downregulation of the insulin receptor substrate 1 (IRS1) [16]. Moreover, a different study reported that higher expression of miR-144 is significantly associated with occurrence of T2DM in a Swedish population^38^. Of note, a recent meta analysis demonstrated evidence for a role of miR-144 as a potential circulating biomarker of the T2DM [40]. The association between miR-144 and T2DM may involve lipid metabolism. Indeed, experimental studies suggested that miR-144-3p can facilitate adipogenesis both in vitro and in vivo, through the promotion of lipid accumulation, which in turn, promotes adipocyte differentiation and the expression of genes associated with fatty acid synthesis [41]. More studies are warranted to test the hypothesis that changes in lipid metabolism through manipulation of miR-144 expression could have impact in T2DM development and/or progression.

Interestingly, our analyses using MOFA demonstrated that miR-924 was another miRNA shown to be related to T2DM and pre-DM patients whereas miR-943 seemed to be more associated with normoglycemic healthy controls and T1DM. To our knowledge, miR-924 and miR-943 have not previously been reported to be associated with the pathogenesis of DM, but there are few reports about miR-924 and miR-943 function, especially in the setting of cancer. In hepatocellular carcinoma tissues and cells associated with hepatitis B virus infection, it has demonstrated that miR-924 may function as a tumor suppressor [42]. In Caco-2 cell lines, miR-924 is implicated in pathogenesis of inflammatory bowel disease by negatively regulating DEFA5 mRNA and protein expression [43]. On the other hand, in women with breast cancer, it has been observed alterations in miR-943 expression levels, further suggesting their involvement in repair of DNA double-strand breaks through TGF-beta pathway control [44]. Furthermore, in an asthma mouse model, it has been demonstrated that miR-943-3p can negatively regulate the expression of SFRP4, causing accelerated progression of airway remodeling in allergic asthma [45].

Our findings demonstrated that miR-200c is related to T1DM instead of T2DM, differently than previous studies which have shown that certain members of the miR-200 family are dysregulated in T2DM and DM-associated complications including vascular inflammation [46,47]. In addition, miR-200c is one of the most upregulated microRNAs found in heart tissue with cardiomyopathy related to diabetes [48]. Furthermore, in arteries from both DM mice and DM patients, it has been demonstrated that high glucose levels upregulates miR-200c via a mechanism dependent on reactive oxygen species (ROS) in endothelial cells [48,49]. These findings suggest that miR-200c is a possible candidate to mediate DM complications, or at least those related to heart and vasculature. Previously, the miR-497 has been proposed to be a predictor of DM due to its upregulation during the acquisition of insulin resistance [50]. Our findings corroborate with this hypothesis by demonstrating that miR-497 expression is associated with pre-DM in the MOFA. Remarkably, miR-497 targets the insulin-like growth factor receptor-1 (IGF-1R) [51] and is upregulated in Goto-Kakizaki rats (which develop T2DM) compared with normal Wistar rats [52].

Our integrative analysis demonstrated that *PLXNC1*, *TMED10*, *MXI1*, *TNFRSF10A* expression profiles are associated with both pre-DM and T2DM. Expression of some of these genes (*IL6R*, *WNK1*, *GRN*) has been previously reported to correlate with metabolic disorders. High expression of *IL6R* has been detected in the serum of patients with T2DM, in diabetic rat pancreatic tissues and in glucose-treated INS-1E cell lines [53]. Furthermore, *WNK1* is considered a mediator of insulin‐stimulated *GLUT4* trafficking since it promotes cell surface abundance of *GLUT4 [54]*. Finally, progranulin is a protein attribute for *GRN* gene and its high serum levels is linked with visceral obesity and T2DM patients [55].

The gene expression of *LSR* and *DLC1* were related to healthy controls and T1DM in the MOFA results, but previous reports suggest a relationship between both of these genes and T2DM. For instance, dysregulation of *LSR* is a common characteristic in the liver of mice with obesity associated with T2DM [56]. Moreover, *DLC*1 is involved in the differentiation of white and brown adipocytes in mouse embryonic fibroblasts, constituting an important component of the metabolic dysfunction which can cause obesity and T2DM [57].

Our study has several strengths. We examined data on a relatively high number of samples which had paired mRNA and miRNA analysis. In addition, we used MOFA as a statistical tool to combine data on mRNA and miRNA data. An important limitation is the lack of biological validation, and a low number of paired samples using different platforms. The batch effects can be corrected for samples comparison. However, it is a mathematical procedure and does not fit the experimental design to explain a biologic effect. Although, the data was corrected by the mean and the variance, there still have co-confounders variables that, the mathematic protocol cannot completely modelled. We deal with this limitation throughout the work and knowning the importance of this exploratory analysis. Nevertheless, this analysis could drive to new insights in the field, and new approaches and validation are required. Regardless, the results presented here contribute to the field as it described a signature able to characterize distinctions between T2DM, T1DM, and pre-DM, identifying several candidate targets for future mechanistic studies focused on elucidation of immunopathogenesis aspects of diabetes.

## Supporting information

S1 File Excel sheet with DEGs comparisons. (XLSX)

S1 Fig Principal component analysis of mRNA dataset. (A) uncorrected (B) corrected, in both datasets patients are depicted by batch in the top, gender in the middle and by group (T1DM, T2DM, preDM and Controls) in the bottom.
(PNG)

S2 Fig Principal component analysis of miRNA dataset. (A) uncorrected (B) corrected, in both datasets patients are depicted by batch in the top, gender in the middle and by group (T1DM, T2DM, preDM and Controls) in the bottom.
(PNG)

S1 Dataset (TXT)

S2 Dataset (TXT)
