# Supplementary figures and images for: In silico transcriptional analysis of mRNA and miRNA reveals unique biosignatures that characterizes different types of diabetes
Source: PLoS One. 2020 Sep 21;15(9):e0239061. doi: 10.1371/journal.pone.0239061 (PMC7505453; doi:10.1371/journal.pone.0239061)

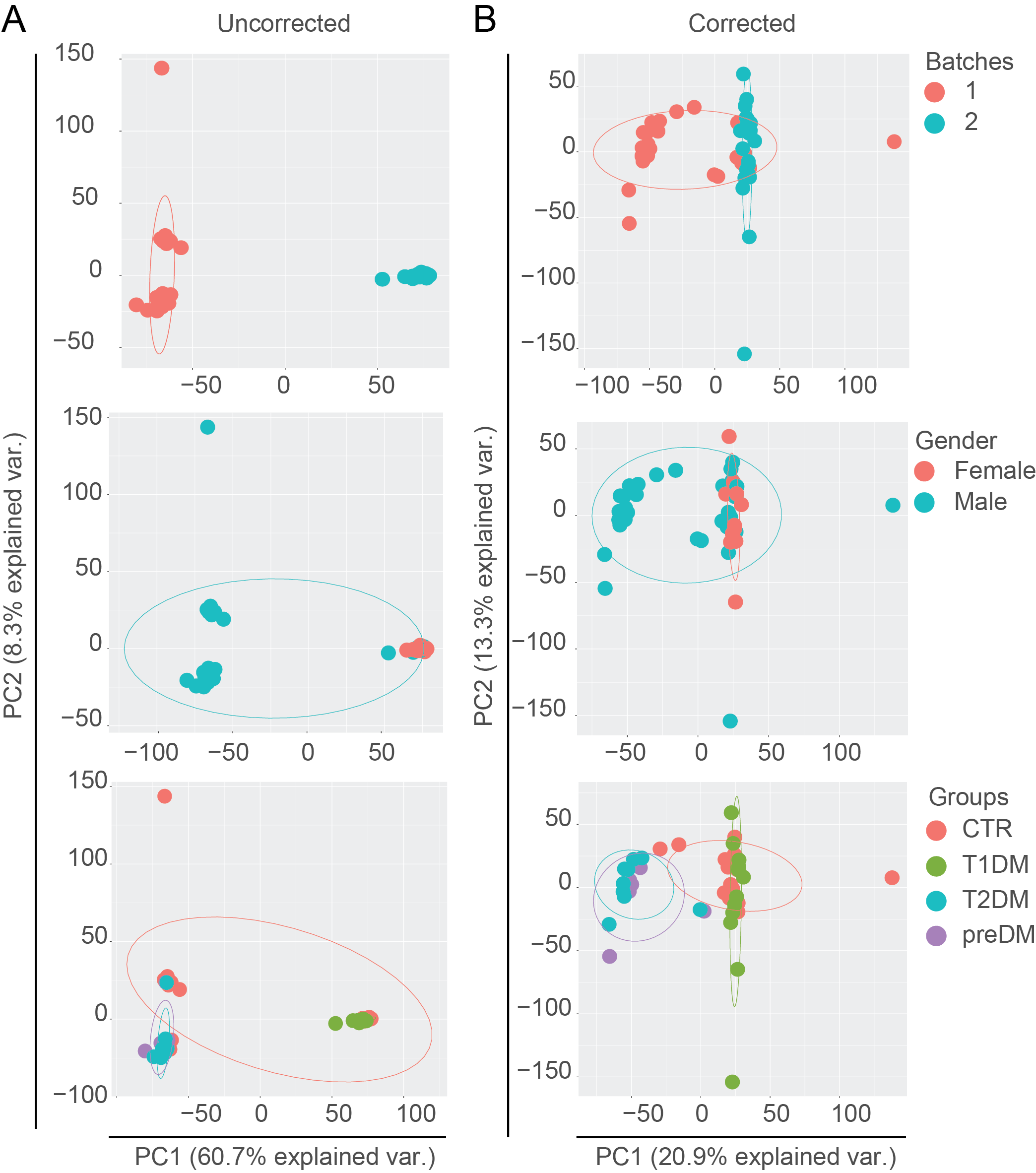

Supplement: S1 Fig — (A) uncorrected (B) corrected, in both datasets patients are depicted by batch in the top, gender in the middle and by group (T1DM, T2DM, preDM and Controls) in the bottom. (PNG) [file pone.0239061.s002.png]

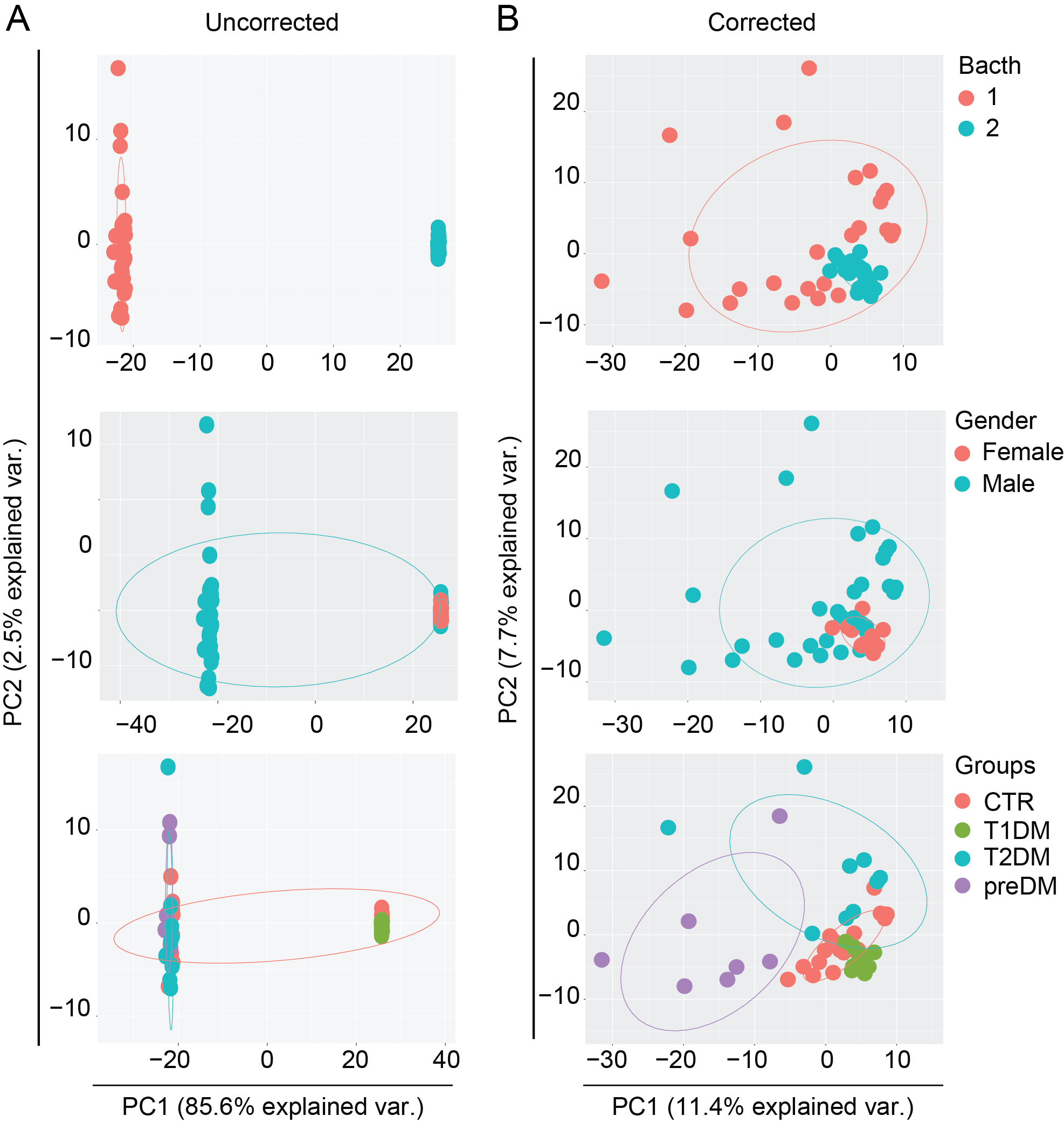

Supplement: S2 Fig — (A) uncorrected (B) corrected, in both datasets patients are depicted by batch in the top, gender in the middle and by group (T1DM, T2DM, preDM and Controls) in the bottom. (PNG) [file pone.0239061.s003.png]
